# The serum IgG antibody level as a biomarker for clinical outcome in patients with cerebral sparganosis after treatment

**DOI:** 10.3389/fimmu.2023.1158635

**Published:** 2023-03-27

**Authors:** Haijie Xiang, Jie Wang, Dandan Tan, Ying Xiong, Pengcheng Huang, Yu Shen, Yun Xu, Zhihong Gong, Fei Hu, Chunhua Xu, Jie Wu, Wei Liu, Junpu Liu, Hui Wan, Daojun Hong, Huiqun Xie

**Affiliations:** ^1^ Department of Neurology, The First Affiliated Hospital of Nanchang University, Nanchang, China; ^2^ Clinical Department, Jiangxi Provincial Institute of Parasitic Disease, Nanchang, China; ^3^ Department of Neurosurgery, The First Affiliated Hospital of Nanchang University, Nanchang, China; ^4^ Department of Neurosurgery, Guangdong 999 Brain Hospital, Guangzhou, China; ^5^ Department of Outpatient, The Nanchang City First Hospital, Nanchang, China

**Keywords:** cerebral sparganosis, praziquantel, surgery, clinical outcome, IgG antibody

## Abstract

**Introduction:**

Cerebral sparganosis is a rare parasitic infection of the brain tissue. The remission of MRI change and clinical symptom has been used to evaluate the therapeutic effect. However, there is no study to correlate the serum IgG antibody level of sparganum to the prognosis of disease after treatment.

**Methods:**

87 patients with cerebral sparganosis were collected from three medical centers. Clinical symptoms and MRI changes were evaluated at 12 months after initial treatment, and serum IgG antibody level of sparganum was evaluated at 2, 6, and 12 months after treatment. The positive cut-off value was based on 2.1 times the optical density (OD) of negative control. The index value was defined as the sample OD divided by the cut-off value.

**Results:**

Among the 87 patients after treatment, 71 patients had good clinical outcomes, and 16 had poor clinical outcomes. The area under the curve (AUC) showed that the index value measured at 12 months after treatment had the best prediction effect, with a value of 2.014. In the good-outcome group, the index values were less than 2.014 in all 71 patients, and only 8 patients had mildly enhanced residual lesions on MRI. In the poor-outcome group, the index values were more than 2.014 in all 16 patients, and all patients still showed significantly enhanced lesions on MRI. Compared with poor-outcome patients, only 2 patients with good outcomes had disease recurrence after treatment.

**Discussion:**

This study provided evidence that the serum IgG antibody level of sparganum was a promising biomarker to evaluate the prognosis of patients with cerebral sparganosis after treatment.

## 1 Introduction

Cerebral sparganosis is a rare parasitic disease caused by the invasion of the plerocercoid larvae into the brain tissue. The main epidemic areas of *Spirometra mansoni* are mainly located in South China and Southeast Asia (1, 2). Previous studies have indicated that the incidence of cerebral sparganosis can account for 25.6% of *Spirometra mansoni* infections (2). Sporadic cases have also been reported in North and South America (3, 4), Europe (5), and Australia (6). The clinical symptoms of cerebral sparganosis include headache, seizures, limb paralysis, aphasia, cognitive impairment, and other focal neurologic defects (7–9). Magnetic resonance imaging (MRI) can reveal some characteristic radiographic features of brain lesions, including aggregated ring-like enhancement, tunnel signs, and migrating lesions (10–12). Collectively, the diagnosis of cerebral sparganosis is based on epidemiological history, characteristic MRI imaging, immunological examination, and pathological changes (13–15).

Surgical removal of the larvae is the principal effective treatment for the disease (7), because the medication used to be considered ineffective against cerebral sparganosis (16). However, recent studies have found that long-term high-dose praziquantel has curative effect in some patients with cerebral sparganosis (17–20). The efficacy evaluation of anthelmintic treatment is mainly based on the remission of clinical symptoms and imaging lesions (7). Nevertheless, some patients can still have the recurrence of cerebral sparganosis despite complete remission of clinical symptoms and imaging lesions after treatment (18). Additionally, the dynamic enhanced MRI is expensive and time-consuming for the follow-up of treatment outcomes. Collectively, these suggest that better biomarkers are needed to evaluate the treatment effect.

Studies have shown that serum IgG antibody level is an important biomarker for epidemiological investigation and sparganosis diagnosis with high sensitivity and specificity, as well as good stability and repeatability (21). A case report showed that the patient with subcutaneous sparganosis had no recurrence after surgery, and their IgG antibody level measured by enzyme-linked immunosorbent assay (ELISA) remained in the normal range at 6 months after surgery (22), indicating the IgG antibody level might be an underlying biomarker for the disease outcome. Currently, there are limited studies on the correlation between the prognosis of cerebral sparganosis after treatment and serum IgG antibody levels. In this study, serum IgG antibody levels were monitored by ELISA in a series of cerebral sparganosis patients who underwent praziquantel or surgical treatment to evaluate the significance of IgG antibody levels as a biomarker for the therapeutic effect of cerebral sparganosis.

## 2 Materials and methods

### 2.1 Study design

Patients with cerebral sparganosis were retrospectively collected in the Jiangxi Provincial Institute of Parasitic Diseases, the First Affiliated Hospital of Nanchang University, and Guangdong 999 Brain Hospital. The principal purpose of this study was to evaluate whether serum anti-sparganum IgG antibody levels could reflect the efficacy and prognosis of the disease treatment. At the end of 2012, our three medical centers established the diagnosis and treatment workflow of cerebral sparganosis. Therefore, the clinical information was comparatively complete in this study.

### 2.2 Patients grouping and follow-up

Patients with cerebral sparganosis treated in the three hospitals from 2013 to 2018 were consecutively collected. The inclusion criteria included: (1) patients presented with neurologic symptoms and signs localized to at least one active lesion in the central nervous system; (2) patients had definite evidence of *sparganum* infection that was proven by immunopositivity to *Spirometra mansoni* antibody in both serum and cerebrospinal fluid (CSF) tests and/or pathological evidence; (3) patients had follow-up of clinical symptoms, cranial MRI and serological IgG antibody level in our centers. The exclusion criteria included: (1) patients lost to follow up; (2) patients with severe cardiopulmonary insufficiency who were not eligible for surgery; (3) patients with severe liver and/or kidney insufficiency leading to contraindication for praziquantel or surgical therapy; (4) patients with other reasons failing to receive surgery or praziquantel treatment.

Baseline demographic and clinical variables were collected as follows: age, sex, epidemiological history, course of the disease, clinical symptom (i.e., seizure, hemiparesis, headache, vertigo, and vomit), cerebral MRI changes (active lesions), serum immunological tests, and treatment (stereotactic aspiration, craniotomy, and praziquantel). Patients were followed up for clinical symptoms and MRI characteristics at 12 months after initial treatment, and the antibody level was followed up at 2, 6, and 12 months after treatment.

The patients were divided into two groups by good or poor outcome. The good outcomes were defined as (1) disappearance of enhanced lesions on cranial MRI; or (2) single nodular lesion with mild enhancement, edema remission, and alleviation of clinical symptoms. The poor outcomes were defined as (1) reappearance of new enhanced lesions on cranial MRI; or (2) persistent enhanced lesions without obvious changes; or (3) the enhanced lesions were reduced, but not meeting the second criterion of the good-outcome group.

### 2.3 Treatment methods

We initially conducted thorough clinical evaluation and medical education about cerebral sparganosis for all patients so that they could choose between praziquantel and surgical therapy. The clinicians provided the treatment options and the final treatment plan according to the willingness of the patients or their legal guardians. The main reasons for choosing long-term high-dose praziquantel treatment were refusal to surgery, lesions located at important functional areas, multiple lesions, and initial tentative therapy. In the praziquantel therapy group, patients were administrated with praziquantel 50 mg/kg/d after meals for 10d per course with a 50d interval, continuing for 8 cycles at most. Surgical treatment would be recommended for patients requiring more than 8 cycles. Surgical treatment included computed tomography-guided (CT-guided) stereotactic aspiration and craniotomy. Praziquantel (50mg/kg/d for 3 days) was routinely given postoperatively to prevent possible residual infection. If the patient developed allergic reactions during praziquantel treatment, intravenous dexamethasone 5mg/d would be administrated for 3-5 days.

### 2.4 Detection of serum IgG antibody level

At 2, 6, and 12 months after treatment with praziquantel or surgery, 2 ml of serum was collected from patients, and the IgG antibody level was detected by indirect ELISA using excretory-secretory (ES) antigens of *Spirometra mansoni (* 23). The ELISA test kits were commercially ordered from Shenzhen Combined Biotech (Shenzhen, China), and the tests were conducted in our in-house lab. The serum of healthy samples were as negative controls. The serum dilution was 1:100, 1:200, 1:400, 1:800, 1:1600, and 1:3200 respectively. 100 μl samples were added to each well. The optical density (OD) of each well was measured at the wavelength of 450 nm. According to the manufacturer’s protocol, the positive cut-off value (COV) was 2.1 times the OD value of negative control. If the OD value of negative control was less than 0.1, the OD value of negative control would be set to 0.1. The positive cut-off values were based on 1:100 diluted serum samples. Thus, sample OD values more than or equal to 0.21 were regarded as positive, and those less than 0.21 as negative. Meanwhile, the serum antibody titer also was calculated as the highest dilution of the serum sample the cut-off value was still greater than 0.21.

### 2.5 Statistical analysis

All statistical analyses were processed by GraphPad Prism Software (GraphPad Prism 9, GraphPad Software Inc.), and p< 0.05 was considered statistical significance. Categorical variables were given as count (percentage). Continuous variables were presented as mean ± standard deviation $\left(\bar{x}\pm sd\right)$ . Comparison between two groups was performed by Mann Whitney test, Chi-squared test, Yates’ continuity corrected Chi-square test, or Fisher’s exact test. Time-dependent receiver operating characteristic (ROC) curve analysis was used to investigate the predictive accuracy of the level of IgG antibody. The area under the curve at different months after treatment was used to measure predictive accuracy. For statistical analysis, the parameter of Y-axis in ROC curve would be converted to the index value (1:100 diluted sample OD divided by cut-off value), instead of using the raw OD value.

### 2.6 Ethical approval and patient informed consent

All patients’ clinical data were anonymous. Eligible patients were approached and informed consent obtained prior to their participation in the study. The Ethics Review Committee of the Jiangxi Provincial Institute of Parasitic Diseases reviewed and approved this study (ethical approval number 2018010). We followed the human experimentation guidelines of the Jiangxi Provincial Institute of Parasitic Diseases in the conduct of the study.

## 3 Results

### 3.1 Treatment options

A total of 87 patients with cerebral sparganosis were enrolled. Fifty-two patients were treated with praziquantel, and 35 patients underwent surgery (**Table 1**). The treatment of patients in the three centers was listed in **Table 2**. There were 32 females and 55 males with a mean age of 27.21 ± 16.00 years and a course of disease ranging from 3 days to 30 years. Among the 87 patients, 15 (17.2%) patients drank uncooked water, 10 (11.5%) patients had a history of eating undercooked frogs that probably had been infected with *sparganum*, and the remaining 62 (71.3%) patients had no clear epidemiological history. There were significant differences in age, disease duration (months), seizure, and headache between surgery group and drug treatment group (**Table 1**). However, the final binary logistic regression model showed no independent variable was associated with the treatment outcome. The efficacy between surgery group and praziquantel group was not significantly different in clinical features, radiological changes, and IgG antibody level (**Table 1** and **Table S1**).

**Table 1:** Baseline characteristics and prognosis in patients with different treatments.

Variables	Praziquantel treatment (n = 52)	Surgical treatment (n = 35)	PValue^a^
Baseline characteristics			
Age (years)	23.00 (16.25, 40.75)	18.00 (13.00, 34.00)	0.048
Male	53.8% (28/52)	74.3% (26/35)	0.054
Disease duration (months)	6.00 (2.00, 21.25)	24.00 (3.00, 48.00)	0.005
Clinical features			
Epidemiological history	30.8% (16/52)	25.7% (9/35)	0.609
Seizure	63.5% (33/52)	97.1% (34/35)	< 0.001
Hemiparesis	36.5% (19/52)	22.9% (8/35)	0.176
Headache	32.7% (17/52)	11.4% (4/35)	0.044
Vertigo	15.4% (8/52)	2.9% (1/35)	0.128
Vomit	5.8% (3/52)	5.7% (2/35)	0.646
Radiological changes			
Active lesions	100% (52/52)	100% (35/35)	> 0.999
CSF immunopositivity	97.9% (46/47)	100% (25/25)	0.747
Index value (OD/COV)	4.362 (3.61, 6.61)	3.814 (2.75, 4.81)	0.073
Prognosis			
Clinical features			
With clinical symptoms	46.2% (24/52)	57.1% (20/35)	0.314
Seizure	26.9% (14/52)	45.7% (16/35)	0.071
Radiological changes			
Active lesions	34.6% (18/52)	17.1% (6/35)	0.074
Index value (OD/COV)	0.93 (0.47, 1.44)	0.562 (0.38, 1.10)	0.115

CSF, cerebrospinal fluid; OD, optical density; COV, cut-off value.

a Comparison between the two groups, using chi-square test, Yates’ continuity corrected Chi-square test, or Fisher’s exact test as appropriate, except for age, disease duration (months), and index value (OD/COV) comparison using Mann Whitney test. And p < 0.05 was considered statistical significance.

**Table 2:** Treatment of patients with cerebral sparganosis mansoni in three centers.

Centers	Number of cases	Number of cases	
		Praziquantel	Surgery
Jiangxi Provincial Institute of Parasitic Diseases	46	46	0
First Affiliated Hospital of Nanchang University	31	6	25
Guangdong 999 Brain Hospital	10	0	10
Total	87	52	35

### 3.2 Demographic and baseline characteristics

Before treatment, all 87 patients with cerebral sparganosis presented with clinical symptoms of central nervous involvement, such as seizures (77.0%), hemiparesis (31.0%), headache (24.1%), dizziness (10.3%), vomiting (5.7%), and so on. Cranial MRI examination showed active lesions, including aggregated ring enhancement, tunnel signs, and migrating lesions, mainly located in the parietal lobe (n = 30), frontal lobe (n = 24), temporal lobe (n = 18), and occipital lobe (n = 6). A total of 71 patients had good clinical outcomes, and 16 patients had poor clinical outcomes. The two groups had no significant differences in demographic data, clinical characteristics, imaging changes, and IgG antibody level before treatment (**Table 3** and **Table S2**).

**Table 3:** Baseline characteristics of patients with different outcomes before treatment.

Variables	Good outcome (n = 71)	Poor outcome (n = 16)	PValue^a^
Age (years)	20.00 (16.00, 40.00)	18.00 (16.25 29.50)	0.581
Male	64.8% (46/71)	50.0% (8/16)	0.271
Disease duration (months)	12.00 (2.00, 24.00)	24.00 (4.00, 48.00)	0.152
Clinical features			
Epidemiological history	33.8% (24/71)	6.25% (1/16)	0.058
Seizure	74.6% (53/71)	87.5% (14/16)	0.438
Hemiparesis	32.4% (23/71)	25.0% (4/16)	0.781
Headache	26.8% (19/71)	12.5% (2/16)	0.378
Vertigo	11.3% (8/71)	6.3% (1/16)	> 0.999
Vomiting	5.6% (4/71)	6.3% (1/16)	0.618
Radiological changes			
Active lesions	100% (71/71)	100% (16/16)	> 0.999
CSF immunopositivity	98.4% (60/61)	100% (11/11)	> 0.999
Index value (OD/COV)	4.25 (3.21, 6.01)	4.49 (3.57, 6.50)	0.357

CSF, cerebrospinal fluid; OD, optical density; COV, cut-off value.

a Comparison between the two groups, using chi-square test, Yates’ continuity corrected chi-square test, or Fisher’s exact test as appropriate, except for age, disease duration (months), and index value (OD/COV) comparison using Mann Whitney test. And p < 0.05 was considered statistical significance.

### 3.3 Clinical outcomes

At 12 months after initial treatment, 57.7% (41/71) of the patients in the good-outcome group showed complete remission of their clinical symptoms, but 40.9% (29/71) still had mild symptoms. In contrast, 93.8% (15/16) of patients in the poor-outcome group showed symptom fluctuation or exacerbation. There was a significant difference between the two groups (40.9%vs93.8%; p<0.001, Yates’ continuity corrected Chi-square test) (**Table 4**).

**Table 4:** Comparison of patients with different prognoses after treatment.

Variables	Good outcome (n = 71)	Poor outcome (n = 16)	PValue^a^
Treatment			
Surgery	40.9% (29/71)	37.5% (6/16)	0.805
Praziquantel	59.2% (42/71)	62.5% (10/16)	0.805
Clinical features			
With clinical symptoms	40.9% (29/71)	93.8% (15/16)	< 0.001
Seizure	26.8% (19/71)	68.8% (11/16)	0.004
Radiological changes			
Active lesions	11.3% (8/71)	100.0% (16/16)	< 0.001
Index value (OD/COV)	0.58 (0.37, 1.01)	4.26 (3.44, 5.15)	< 0.001
Recurrence	2.8% (2/71)	100.0% (16/16)	< 0.001

OD, optical density; COV, cut-off value.

a Comparison between the two groups, using chi-square test, Yates’ continuity corrected chi-square test, or Fisher’s exact test as appropriate, except for index value (OD/COV) comparison using Mann Whitney test. And p < 0.05 was considered statistical significance.

**Figure 1:**
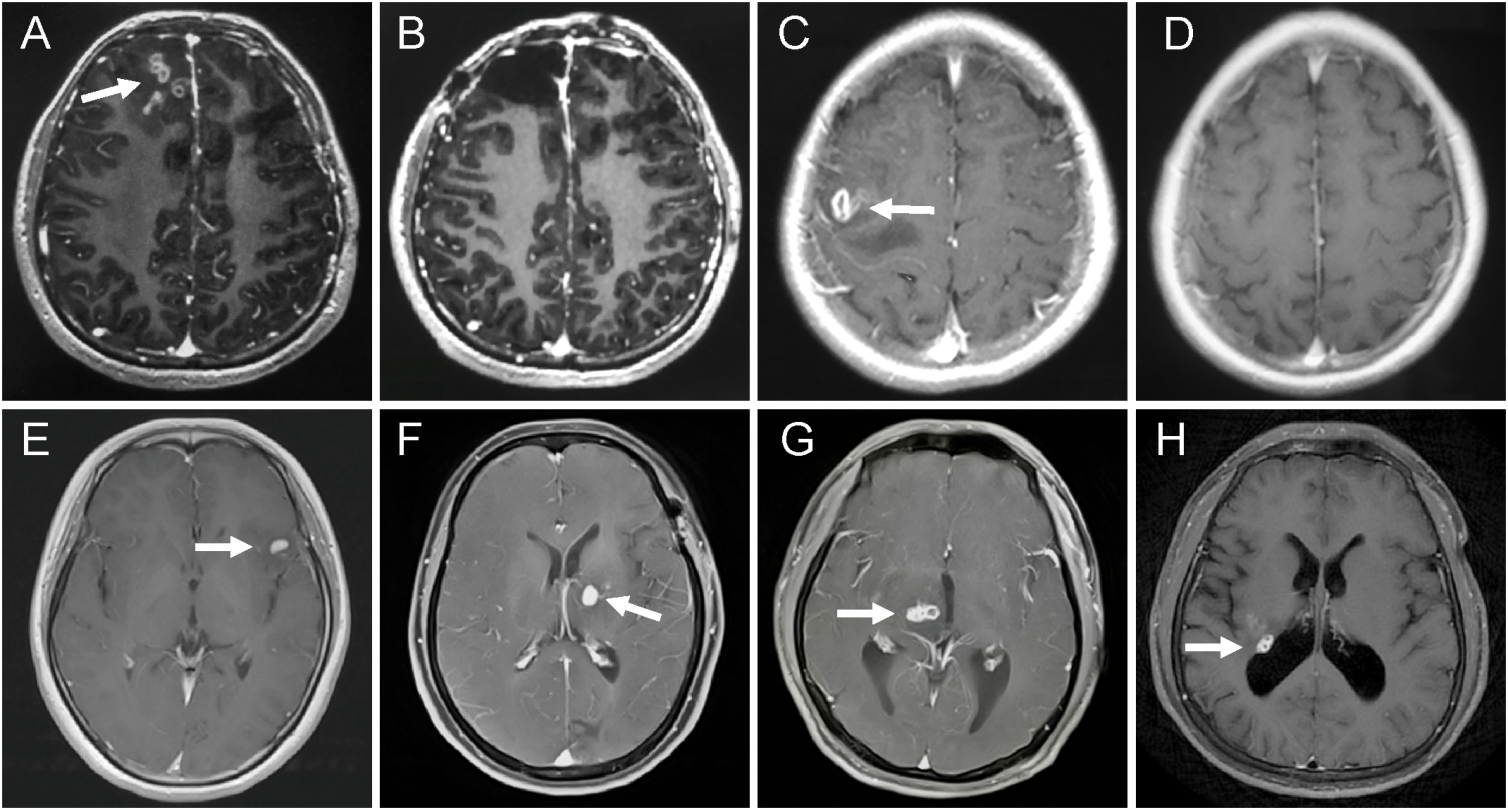
MRI changes of the patients with different treatment options and outcomes. **(A–D)** patients with good outcomes. **(A)** axial T1-weighted images revealed aggregated bead-shaped enhancement in the subcortical area of the right frontal lobe. **(B)** the active lesions were removed by surgery. **(C)** axial enhanced MRI showed nodular enhanced lesions in the left temporal lobe. **(D)** the enhanced lesions completely disappeared after 12 months with high-dose praziquantel. **(E–H)** patients with poor outcomes. **(E)** T1-weighted image demonstrating a crescent-shaped enhanced lesion in the left temporal lobe. **(F)** a new enhanced lesion reoccurred in the left basal ganglia at six months after surgery. **(G)** axial T1-weighted image showed a tunnel sign in the right thalamus. **(H)** T1-weighted image showed a round enhanced lesion in the right lateral posterior ventricle at 12 months after praziquantel treatment.

At 12 months after initial treatment, 88.7% (63/71) of patients in the good-outcome group showed the disappearance of active lesions on cranial MRI (**Figures 1A, B** for surgical treatment; **Figures 1C, D** for praziquantel treatment). The remaining 11.3% (8/71) of patients had residual lesions but all showed a shrunk solitary nodular lesion with mild enhancement. In contrast, 100% (16/16) of patients in the poor-outcome group still showed active lesions on cranial MRI, of which 43.8% (7/16) had larvae migration; 37.5% (6/16) had no changes in the lesions; 12.5% (2/16) had new enhancement lesions; and 6.3% (1/16) had enlarged lesions after treatment (**Figures 1E, F** for surgical treatment; **Figures 1G, H** for praziquantel treatment). The MRI changes between the two groups had a significant difference (11.3% vs100%; p<0.001, Fisher’s exact test) (**Table 4** and **Table S3**).

### 3.4 IgG antibody changes

**Figure 2:**
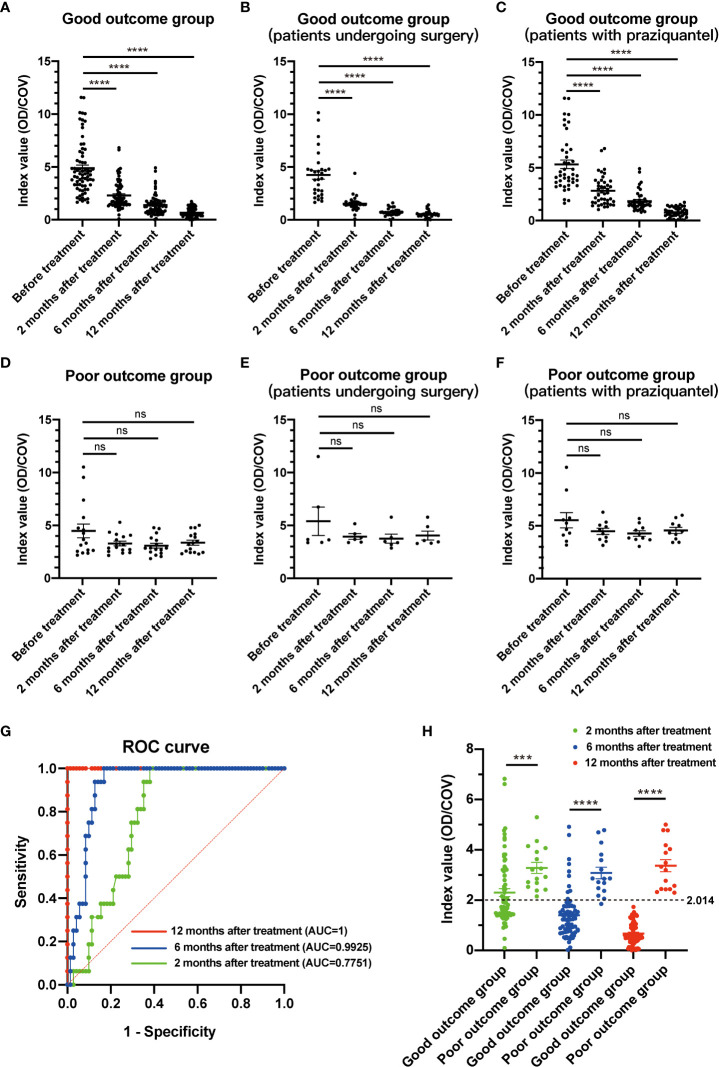
The index value changes of serological examination in patients with different clinical outcomes. **(A)** In the good-outcome group, the index value of IgG antibody showed significant decrease differences between before and after treatment. **(B)** In the good-outcome group of patients who underwent surgery, the index value of IgG antibody showed a significant decrease between before and after treatment. **(C)** In the good-outcome group of patients with praziquantel, the index value of IgG antibody showed a significant decrease between before and after treatment. **(D)** In the poor-outcome group, the index value of IgG antibody showed no significant differences between before and after treatment. **(E)** In the poor-outcome group of patients who underwent surgery, the index value of IgG antibody showed no significant differences between before and after treatment. **(F)** In the poor-outcome group of patients with praziquantel, the index value of IgG antibody showed no significant differences between before and after treatment. **(G)** The area under the curve (AUC) showed that the index value measured at 12 months after treatment had the best prediction effect. **(H)** In the good-outcome group, the index value of IgG antibody was less than 2.014 in all 71 patients at 12 months after treatment. In the poor outcome group, the index value was more than 2.014 in all 16 patients at 12 months after treatment. For all figures: one-way ANOVA with Tukey’s multiple comparisons test, ***p < 0.001; ****p < 0.0001; ns, no significant difference. Error bars indicate the SEM.

At 2, 6, and 12 months after treatment, the index value of IgG antibody level in patients with good outcomes significantly decreased (**Figure 2A**), while those with poor outcomes showed no significant differences (**Figure 2D**). In the good-outcome group of patients undergoing surgery or with praziquantel, the index value of IgG antibody showed significant decrease differences between before and after treatment (**Figures 2B, C**), while in the poor-outcome group of patients undergoing surgery or with praziquantel, the index value of IgG antibody showed no significant differences (**Figures 2E, F**). The area under the curve (AUC) showed that the index value (OD/COV) measured at 12 months after treatment had the best prediction effect, with a value of 2.014 (**Figures 2G, H**). All patients with good outcomes at 12 months after treatment had an index value less than 2.014, while all patients with poor outcomes at 12 months had an index value more than 2.014 (**Figure 2H**). Consequently, there was also a significant difference in the serum anti-sparganum IgG antibody level between the two groups (p<0.001, Mann Whitney test) (**Table 4**).

Most patients (71/87, 81.6%) with cerebral sparganosis had a good prognosis after praziquantel or surgical therapy. In the good-outcome group,11.3% (8/71) patients showed mildly residual lesions on MRI, while 100% (71/71) had an index value less than 2.014, suggesting that serum IgG antibody level might be a more sensitive and incipient indicator than MRI changes. In the poor-outcome group, one patient (1/16, 6.3%) showed symptom alleviation after treatment, and all patients had an index value more than 2.014, indicating that *Spirometra mansoni* had not been completely cleared.

At 12 months after initial treatment, only 2 patients with an index value less than 2.014 (2/71, 2.8%) were observed to have a recurrence of cerebral sparganosis after treatment, but 16 patients with an index value more than 2.014 (16/16, 100%) still had active or migrating lesions that could be considered as a type of recurrence of cerebral sparganosis. Consequently, there was a significant difference in the recurrence of cerebral sparganosis between the two groups (2.8% vs 100%; p<0.001, Fisher’s exact test) (**Table 4** and **Table S3**).

## 4 Discussion

Cerebral sparganosis is an unusual parasitic illness caused by *Spirometra mansoni (* 5). Larvae will migrate and damage brain tissue, resulting in neurological dysfunction mediated by inflammation (9, 24). Resection of the worm, such as craniotomy or CT-guided stereotactic aspiration is the preferred method for the treatment of cerebral sparganosis (25). Recent studies have also revealed that patients with cerebral sparganosis can also be successfully treated with long-term high-dose praziquantel (25-75 mg/kg/d) (16–20). In this study, our data also confirmed that long-term high-dose praziquantel showed similar efficacy with surgery, although some baseline characteristics had differences between surgery group and drug treatment group.

Currently, the treatment outcome of cerebral sparganosis is generally based on the changes in clinical presentations and cerebral MRI lesions, but these criteria involve some degree of subjectivity. A previous study found that cerebral sparganosis recurred in some cured patients who showed complete remission of clinical symptoms and imaging lesions after treatment (18), indicating that clinical presentation and imaging improvement as efficacy endpoints had certain limitations. The purpose of this study was to find an objective laboratory biomarker to predict the treatment outcome of cerebral sparganosis.

In this study, 100% of the patients had an index value less than 2.014 in the good-outcome group, although 11.3% showed slightly residual lesions on enhanced MRI. Therefore, the IgG antibody level tested by ELISA showed better sensitivity than enhanced MRI to evaluate the clinical outcome. In the poor-outcome group, follow-up monitoring of the IgG antibody level showed a strong positive reaction (more than 2.014), indicating that the larvae might be in a dormant or drug-resistant state that caused difficulty to be eliminated (26). In addition, for patients whose index value remained more than 2.014 after treatment, we followed up on imaging and found that there were still active lesions. Some of them even developed new symptoms or new enhanced lesions. Our study showed that patients with an index value less than 2.014 (2/71, 2.8%) had a significantly lower incidence of the recurrence of cerebral sparganosis than patients with an index value more than 2.014. Enhanced MRI was expensive and time-consuming, while ELISA was more economical and time-saving to detect sparganosis (21). Therefore, the IgG antibody level tested by ELISA can be applied to evaluate the prognosis of cerebral sparganosis, and compensate for the limitations of symptom and imaging changes.

The parasite secreted a variety of antigens, such as ES antigens, into the peripheral blood circulation of patients in the early stage of infection, and induced strong antibody responses. Therefore, ELISA is the most commonly used method for the diagnosis of sparganosis. Previous studies reported that the sensitivity and specificity of ES antigen ELISA in the diagnosis of *sparganum* infections were up to 100% and 100%, respectively (23, 27). However, there was no multicenter large cohort study investigating important laboratory biomarkers for predicting the efficacy of cerebral sparganosis treatment. A single-center, small cohort study, in which examined changes in peripheral blood eosinophil counts during follow-up, concluded that eosinophil absolute counts cannot be used as an effective prognostic indicator (20). In our study, we found that the detection of serological IgG antibody was an excellent choice for judging patient prognosis.

In the good-outcome group, patients’ serum antibody level gradually decreased, although the antibody still could be slightly detectable in some patients after complete recovery. There were also some cases that the antibody level could return or decrease to the normal range after treatment (22, 28). However, no studies have followed long-term antibodies after treatment in patients with sparganosis. Cui et al. found that slightly infected mice still had anti-sparganum antibodies at 18 weeks after infection, suggesting that ELISA can also be used for long-term serum follow-up of low-level infection sparganosis (29). Previous epidemiological studies have found that many seropositive subjects showed no clinical evidence of sparganosis, indicating that these subjects might have occult infections or previous infections (30–32). This indicated that patients with sparganosis who had achieved clinical cure could maintain a long-term low-titer serum antibody, but its specific mechanism was still unclear. Our results showed that serum IgG antibody level might play an important role in the follow-up of patients with cerebral sparganosis after treatment, especially when the clinical symptoms and imaging had improved.

In general, if the index value of IgG antibody level is less than 2.014, the prognosis usually will be favorable and enhanced MRI will not be required. However, when the index value is more than 2.014, follow-up will be necessary to pay attention to whether new lesions will occur. Collectively, we commended some indicators for successful treatment of cerebral sparganosis: (1) the patients had no symptoms or the frequency of seizure significantly decreased; (2) enhanced MRI showed no active lesions; (3) the index value of IgG antibody was less than 2.014. The following indicators were considered treatment failure: (1) clinical symptoms showed no improvement or new symptoms occurrence, or the frequency of seizures increased; (2) enhanced MRI showed the presence of persistent lesions or new lesions; (3) the index value of antibody was more than 2.014.

Our study had some limitations. First, this was a retrospective, non-randomized study. Since patients with cerebral sparganosis were relatively rare, more cases need to be collected from more centers for further confirmation. Second, this study only followed up on the efficacy for 12 months after treatment. A longer follow-up study should be conducted, although it was difficult to evaluate the effectiveness and prognosis over a longer period. Third, the index value might only be applicable to the ELISA kits of Shenzhen Combined Biotech. According to the dilution titer, the index value approximately corresponded to the 1:200 IgG antibody titer, which was more valuable for clinical guidance.

## 5 Conclusion

In summary, this study provided evidence that the serum anti-sparganum IgG antibody level could be used as a serum biomarker to assess the treatment effect of cerebral sparganosis. If the index value of antibody is less than 2.014, the prognosis usually will be favorable and enhanced MRI will not be required. However, when the index value of antibody is more than 2.014, follow-up will be necessary to pay attention to whether new lesions will occur. We hoped that our findings could have some enlightenment for the medication of cerebral sparganosis and lay a foundation for the prognosis evaluation of cerebral sparganosis in the future with larger samples, randomized, and prospective studies.

## Data availability statement

The original contributions presented in the study are included in the article/**Supplementary Material**. Further inquiries can be directed to the corresponding authors.

## Ethics statement

The studies involving human participants were reviewed and approved by The Ethics Review Committee of the Jiangxi Provincial Institute of Parasitic Diseases. Written informed consent to participate in this study was provided by the participants’ legal guardian/next of kin.

## Author contributions

DH, HXie, CX, JWu, and YXu conceptualized and administrated the study. DH, HXie, ZG, JL, and FH completed the methodology. HXia, PH, YXi, DT, and YS handled the software. DH, HXie, JWu, HW, and CX validated the study. DH, HXie, YXu, and JL did the formal analysis. HW, YXu, CX, JL, and JWu did the investigation. DH, HXie, YXu, and JL analyzed the data. HXia, JWa, HXie, and DH wrote the manuscript. DH, HXia, CX, JWu, and HXie contributed to the manuscript revision. DH, HXie, JWu, and CX visualized the study. DH, HXie, HW, ZG, and FH supervised the study. DH, and HX: Funding acquisition. All authors contributed to the article and approved the submitted version.

## Supplementary material

Supplementary File 1 .

Supplementary Table 1 m, male patient; f, female patient. N, no; Y, yes; +, positive; -, negative; NT, not tested; CSF, cerebrospinal fluid; OD, optical density; COV, cut-of value (0.21); Epidemiological history indicates that patients were infected with Spirometra by drinking uncooked water, or had a history of swallowing raw frogs that probably have been infected with sparganum.

Supplementary Table 2 m, male patient; f, female patient. N, no; Y, yes; +, positive; -, negative; NT, not tested; P, Praziquantel; S, Surgery; CSF, cerebrospinal fluid; OD, optical density; COV, cut-of value (0.21); Epidemiological history indicates that patients were infected with Spirometra by drinking uncooked water, or had a history of swallowing raw frogs that probably have been infected with sparganum.

Supplementary Table 3 N, no; Y, yes; -, negative; P, Praziquantel; S, Surgery; CSF, cerebrospinal fluid; OD, optical density; COV, cut-of value (0.21).
